# Old and New Approaches to Animal Cognition: There Is Not “One Cognition”

**DOI:** 10.3390/jintelligence8030028

**Published:** 2020-07-02

**Authors:** Juliane Bräuer, Daniel Hanus, Simone Pika, Russell Gray, Natalie Uomini

**Affiliations:** 1 Max Planck Institute for the Science of Human History, Department of Linguistic and Cultural Evolution, Kahlaische Strasse 10, 07745 Jena, Germany; gray@shh.mpg.de (R.G.); traduck@gmail.com (N.U.); 2 Department of General Psychology, Friedrich-Schiller-University, Am Steiger 3, 07743 Jena, Germany; 3 Max Planck Institute for Evolutionary Anthropology, Department of Developmental and Comparative Psychology, Deutscher Platz 6, 04103 Leipzig, Germany; hanus@eva.mpg.de; 4 Institute of Cognitive Science, Comparative BioCognition, University of Osnabrück, Artilleriestrasse 34, 49076 Osnabrück, Germany; simone.pika@uni-osnabrueck.de

**Keywords:** animal cognition, comparative psychology, comparative cognition, animal psychology, animal minds, cognitive evolution

## Abstract

Using the comparative approach, researchers draw inferences about the evolution of cognition. Psychologists have postulated several hypotheses to explain why certain species are cognitively more flexible than others, and these hypotheses assume that certain cognitive skills are linked together to create a generally “smart” species. However, empirical findings suggest that several animal species are highly specialized, showing exceptional skills in single cognitive domains while performing poorly in others. Although some cognitive skills may indeed overlap, we cannot a priori assume that they do across species. We argue that the term “cognition” has often been used by applying an anthropocentric viewpoint rather than a biocentric one. As a result, researchers tend to overrate cognitive skills that are human-like and assume that certain skills cluster together in other animals as they do in our own species. In this paper, we emphasize that specific physical and social environments create selection pressures that lead to the evolution of certain cognitive adaptations. Skills such as following the pointing gesture, tool-use, perspective-taking, or the ability to cooperate evolve *independently* from each other as a concrete result of specific selection pressures, and thus have appeared in distantly related species. Thus, there is not “one cognition”. Our argument is founded upon traditional Darwinian thinking, which—although always at the forefront of biology—has sometimes been neglected in animal cognition research. In accordance with the biocentric approach, we advocate a broader empirical perspective as we are convinced that to better understand animal minds, comparative researchers should focus much more on questions and experiments that are ecologically valid. We should investigate nonhuman cognition for its own sake, not only in comparison to the human model.

## 1. Current Hypotheses on Animal Cognition

Some of the most enduring questions in contemporary behavioral science concern which cognitive skills humans share with other animal species and which are uniquely human (Premack and Woodruff 1978; Byrne 1996; Tomasello 2019). One prevalent approach to this question is the comparative approach, which pinpoints similarities and differences between human and nonhuman animals to then draw informed inferences about the evolution of human behavior and cognition (Heyes and Huber 2000; Tennie et al. 2009; Borrego 2017). The term “cognition” (see Box 1) refers to “adaptive information processing in the broadest sense, from gathering information through the senses to making decisions and performing functionally appropriate actions, regardless of the complexity of any internal representational processes that behavior might imply” (Shettleworth 2000, p. 43). According to this definition, animals receive inputs to their brain through, e.g., vision, hearing, touch, smell, taste, electric fields, air currents, or magnetic fields. The brain processes these inputs and controls behaviors. Behavior is the result of an animal’s decision to act on the inputs received. This definition of cognition encompasses all possible inputs and actions that are the result of mental processes. In this paper, we rely on this definition without distinguishing between more or less complex or sophisticated forms of cognition, as that would involve a judgment of what qualifies as “sophisticated”, which is what we want to avoid here, as we argue below. We are aware that animals may often use simple mechanisms to solve their social and physical problems, reserving the more complex mechanisms for situations in which the simpler mechanisms do not work. The important point for us is that individuals show flexible behavior and do not rely on innate or learned strategies only. In this paper, we show how this broad definition of cognition is at odds with narrow views that are still prevalent in viewing cognition as “one cognition”, in contrast to the biocentric perspective that focuses on each species in its own evolutionary history, which we support here.

Box 1 **Definitions used in this article.**

**Comparative Psychology**: Investigation of similarities and differences in multiple animal species—including humans—using techniques that encompass everything from observational studies in nature to neurophysiological research in the laboratory (Call et al. 2017; Tomasello and Herrmann 2010).

**Cognition**: Adaptive information processing in the broadest sense, from gathering information through the senses to making decisions and performing functionally appropriate actions, regardless of the complexity of any internal representational processes that behavior might imply (Shettleworth 2000).

**Physical Cognition**: Knowledge of the physical world (Tomasello and Call 1997).

**Social Cognition**: Knowledge of the social world (Tomasello and Call 1997).

**Animal Cognition**: Describes the mental capacities of nonhuman animals and the study of those capacities. The field developed from comparative psychology, including the study of animal conditioning and learning (Shettleworth 2000.)

We now know that cognition comes in many forms across a huge diversity of nonhuman animal species (i.e., Shettleworth 1998; McMillan et al. 2015; Vonk 2016; Allen 2017; Call et al. 2017). The first studies on the complexity of animal minds were carried out by psychologists and traditionally centered on the phenomenon of learning (Kamil 1987; Hanus 2016; Maestripieri 2003), for instance, in a few model species such as rats and pigeons (Beach 1995; Hodos and Campbell 1969; Papini 2002). However, they were ignoring the biological context of behaviors, i.e., their potential adaptive implications (i.e., Kamil 1987). In a pioneering article entitled “A synthetic approach to the study of animal cognition”, Kamil (1987) argued for a broader approach to understanding animal minds and stressed two main points: (1) that the range of investigated skills needs to be expanded—i.e., to study phenomena besides learning, and (2) that researchers should consider ecological factors to situate their findings within a comparative evolutionary framework (Kamil 1987; see also Shettleworth 1983; Bates and Byrne 2007; Bshary et al. 2011).

Since the publication of that article, researchers in the fields of comparative cognition, animal psychology, cognitive archaeology, and cognitive biology (Shettleworth 2009; Fitch et al. 2010) have made considerable advances in tackling different cognitive and behavioral elements that form the layered system of cognition. Consequently, the range of skills and species studied has now significantly increased. In particular, in the last 20 years, there has been a growing interest in these fields (see Shettleworth 2009 for an overview), resulting in increasing citation rates from about 400 citations per year in 2000 to over 4000 citations in 2019 (Table 1). The research in these fields focused on two major challenges animals have to deal with: foraging and maintaining social relationships (Tomasello and Call 1997; Seyfarth and Cheney 2003; Seed et al. 2009). To date, the majority of the studies have examined distinct aspects of physical cognitive skills such as tool-use, memory, future planning, and numerosity, as well as social cognitive skills such as communication, cooperation, and social learning (Call et al. 2017; Shettleworth 1998; Shettleworth 2010; De Waal and Tyack 2009; Reznikova 2007; Wasserman and Zentall 2006; Zentall and Wasserman 2012).

Kamil’s (1987) first suggestion was heeded. However, Kamil’s (1987) second suggestion, to adopt a biocentric view of cognition by considering ecological factors, which is quite natural for biologists, has been at times neglected by psychologists (Sewall 2015; Macphail 1982; Eaton et al. 2018; Vasilieva 2019). We see two main problems that hinder current research in comparative psychology. Firstly, an anthropocentric approach dominates research (Shettleworth 2010). Secondly and relatedly, cognition is viewed as a cluster of skills that evolve together, as in humans. We see that these positions are still prevalent in comparative psychology (Vasilieva 2019).

In particular, psychologists have postulated several prominent hypotheses to explain why certain species are considered “intelligent”. These hypotheses assume that cognitive skills are linked together to create a broad (or universal) instantiation of intelligence, often termed cognitive flexibility (for an overview, see Allen 2017). For instance, the *Social Intelligence Hypothesis*—also termed the Machiavellian Intelligence Hypothesis or Social Brain Hypothesis (Dunbar and Shultz 2007; Humphrey 1976; Jolly 1966; Byrne and Whiten 1988)—seeks to explain the origins of primate intelligence in their sociality. It predicts that natural selection favored those individuals living in complex social environments, such as fission–fusion societies, for their ability to deal with the frequent unpredictable situations that occur in social interactions in such societies. Thus, intelligence is triggered by the demands and complexity of sociality. In a similar vein, the *Cognitive Buffer Hypothesis* predicts that large brains facilitate the construction of behavioral responses to unusual, novel, or complex socioecological challenges. This buffer effect should increase survival rates and favor a longer reproductive life, thereby compensating for the costs of delayed reproduction (Sol 2009). The *Domestication Hypothesis* (Hare and Tomasello 2005; Hare et al. 2012) proposes that selection for reduced aggression in some species, such as in domesticated species like dogs, but also in wild bonobos (*Pan paniscus*), caused a set of cognitive changes, including increases in levels of social tolerance, sensitivity to social cues, cooperation, risk aversion, occurrence of juvenile behaviors, and reduction of spatial memory. Similar to the Social Intelligence Hypothesis, the Domestication Hypothesis argues that a whole suite of cognitive skills was triggered by a single factor. The *Cooperative Breeding Hypothesis* (Burkart et al. 2016; Burkart and van Schaik 2010; Burkart et al. 2009) considers the practice of cooperative breeding to have caused a “cascade” of effects on cognition such as changes in general intelligence, language, prosociality and social tolerance, teaching, and tool-making skill. It proposes that human cognitive abilities were amplified by our prosocial tendencies, again placing sociality at the center of a suite of changes. The *Technical* (or *Physical*) *Intelligence Hypothesis* proposes that great apes’ complex food manipulations using tools caused the emergence of an ability for “flexible plan-building”, which involves a representational ability that could then be applied to living entities like conspecifics (Byrne 1997). This ability allowed great ape ancestors to view other individuals as intentional agents, and thus to engage in social manipulations involving complex planning. Hence, the Technical Intelligence Hypothesis also predicts that tool-use and sociality are linked by a shared ability for flexible action planning.

This brief survey of selected major hypotheses exemplifies the problematic assumption that “intelligence” results from a cluster of cognitive skills that are linked together and are elicited by *single* evolutionary conditions and factors that create a set of selection pressures. All these hypotheses seek to explain why cognitive skills have evolved in particular species, but not in others—there is evidence for all of the hypotheses presented above, but they do not explain the whole picture. The data supporting these hypotheses have been discussed elsewhere (i.e., Burkart et al. 2016). However, cognitive arrays are the result of species-typical adaptions to their *whole* ecological and social environments (Burkart et al. 2016).

Hence, the aim of the current paper is to counteract an overly simplistic reading of these hypotheses by emphasizing the cases that contradict them, showing that cognitive skills are often not linked together.

The related problem which arises from the “one cognition” assumption is that, implicitly or explicitly, the presented hypotheses consider human cognition as the maximum and standard capacity (Vasilieva 2019). This idea is exemplified in cognitive niche construction theory, which considers human cognition to have evolved into the most flexible and adaptable form of intelligence due to a runaway feedback process of cumulative culture and developmental plasticity (Sterelny 2003; Laland et al. 2000). Cognitive abilities of target species are subsequently compared and measured according to whether and how much they match the suite of human abilities. Historically, comparison against human standards was one of the original roles of animal cognition research derived from comparative psychology (Beran et al. 2014; Shettleworth 1998; Kamil 1987; Box 1). As interest in other minds shifted from humans to nonhuman species, methods of human psychology were often transferred to other animals. However, this approach can only produce a restrictive, anthropocentric view of cognitive evolution that ignores the incredible diversity of cognitive skills present in the world (Bates and Byrne 2007). On the contrary, considering each species in its own right—in accordance with traditional Darwinian thinking (Darwin 1859)—allows us to reveal the evolutionary, developmental, and environmental conditions that foster the growth of certain unique abilities in the young of a species, or the convergence of skills shared among species (Griffiths and Stotz 2000; Shettleworth 2009; Shettleworth 2010). The core of this biocentric view is expressed in Darwin’s metaphor: “Nature [selects] only for that of the being which she tends. Every selected character is fully exercised by her, and the being is placed under well-suited conditions of life.” (Darwin 1859, p. 83).

We believe the criticism raised by Shettleworth in 2010 is still valid today: “Although the extent of human–animal cognitive similarity is undoubtedly a key issue for comparative psychology, it sometimes seems the agenda is to support anthropomorphic interpretations” (Shettleworth 2010, p. 2). One example is the investigation of the understanding of the human pointing gesture. There are a plethora of comparative studies in which animals are required to use human gestures—mainly the pointing gesture—to locate hidden food (reviewed by Krause et al. 2018; i.e., Bräuer et al. 2006). From a biocentric point of view, it does not make much sense to ask whether, for example, a nonhuman ape follows a human gesture as this task is not ecologically relevant to the ape. Firstly, humans are not relevant; secondly, although apes produce pointing gestures to communicate with humans in captivity (Leavens et al. 1996; Halina et al. 2018), they rarely inform others about external events in the wild (Burrows et al. 2013). Thirdly, due to their competitive social systems, nonhuman apes would not be expected to inform others about the location of hidden food (Sterck et al. 1997; Wittig and Boesch 2003; Bräuer et al. 2006). Not surprisingly, for biologists, it turned out that apes and most of the animal species tested did not reliably follow the human pointing gesture to locate hidden food unless they were enculturated (i.e., had a lot of intense experience with humans) or belonged to a species that was domesticated. It is interesting that domesticated animals follow the pointing gesture, but it is not surprising that apes have problems doing so, given their social, ecological, and evolutionary backgrounds. Similarly, the studies of language-trained apes (reviewed in Gillespie-Lynch et al. 2014) give us some insight into what these animals are capable of with a large amount of training, although during evolution, there was no selection pressure to communicate with humans. Thus, these studies do not tell us much about apes, but rather about humans’ specially evolved skills such as language (Morgan et al. 2015; Uomini and Meyer 2013; Uomini 2009; Uomini 2014; Uomini 2017; Uomini and Ruck 2018). Instead, these studies can tell us what these species are able to learn about situations they do not encounter naturally. For example, if some chimpanzee individuals can learn such “unnatural” skills as following the human pointing gesture or using a lexigram language, this shows us chimpanzees’ cognitive flexibility and can help us to understand the factors leading to innovation and their ability to learn and generalize. In contrast, the aim of a less anthropocentric approach should be to investigate their natural forms of communication and not to force them to use human-like communication.

In this paper, we discuss two related problems, namely, the assumption that cognition evolves as a cluster of skills as in humans, and the anthropocentric approach, following the criticisms raised by Shettleworth (1998, 2009, 2010). However, despite the subsequent progress noted by Shettleworth (2009), we still consider it necessary to advocate for a broader perspective on cognitive evolution. To be clear, we do not argue against comparative psychology as a valid discipline to gain insights into animal cognition; rather, we argue against *how* comparisons between humans and other animals are carried out by favoring the anthropocentric issues mentioned above. If we want to account for the fascinating variety of animal minds, comparative scientists should focus on skills that are ecologically relevant for a given species (Section 2), as well as skills in which humans are outperformed by other animals (Section 3). Moreover, the experimental operationalization of a research question should be ecologically valid, i.e., using naturalistic situations with relevant test settings that match naturally occurring contexts and—most importantly—the modality must be relevant for the tested species (Section 4). By eschewing the traditional anthropocentric approach and turning our attention to skills that humans either do not excel in or do not possess, we are better positioned to advance the science of animal cognition (Cantlon and Hayden 2017).

## 2. Performing Competently–Performing Poorly: Cognitive Skills Are Not Necessarily Linked Together

To illustrate the pitfalls of the anthropocentric approach, let us briefly consider human cognition as unique (i.e., MacLean 2016; Tomasello 2019; Sterelny 2003) and as the maximum capacity (Pinker 2010; Suddendorf and Busby 2003; Jiang et al. 2018). In this case, we would assume that our closest living relatives, great apes, would show cognitive skills similar to humans, whereas less related species from other clades would underperform. However, numerous empirical findings of the past decades confirm that not only apes but also several other previously underestimated animal species demonstrate unexpected cognitive skills. Several bird species, for instance, show skills comparable to nonhuman primates in tasks concerning object permanence, delay of gratification, causal reasoning, theory of mind, and mental time travel (see Güntürkün and Bugnyar 2016 and McMillan et al. 2015 for reviews). As an example, western scrub jays *(Aphelocoma californica*) hide food caches for future consumption, steal others’ caches, and engage in tactics to minimize the chance that their own caches will be stolen (Dally et al. 2006). They also show spontaneous future planning behavior without reference to their current motivational state (Raby et al. 2007). Similarly, two more recent studies revealed that ravens (*Corvus corax*) seem to plan for the future by saving tools for future use and tokens for future bartering (Kabadayi and Osvath 2017; but see Redshaw et al. 2017 for an alternative interpretation). They also attribute visual access to unseen competitors (Bugnyar et al. 2016). Moreover, in a study on the natural communication abilities of ravens in the wild, Pika and Bugnyar (2011) showed that ravens use an extremely rare form of attention-getters, a communicative ability previously confined to primates only. Furthermore, there is evidence for flexible cognitive skills in fish (see Patton and Braithwaite 2015; Bshary and Brown 2014 for reviews). Examples include the ability to use transitive inference—i.e., to conclude that if A > B and B > C then A > C—in cichlid fish (Grosenick et al. 2007), numerical competence to track shoal size in shoaling fish (Agrillo et al. 2012), updating rules to decide whether and from whom to learn about the location of food sources in nine-spined sticklebacks (Pike and Laland 2010), and interspecific collaborative hunting in coral reef fishes (Vail et al. 2013). Additionally, in reptiles (Matsubara et al. 2017), insects (Feinerman and Korman 2017; Webb 2012) and nonprimate mammals, there are new findings of unexpected cognitive skills such as social learning and face discrimination in domestic pigs (Veit et al. 2017; Wondrak et al. 2018) or size and shape discrimination in horses (Tomonaga et al. 2015). Data from social carnivores show that they are capable of “numerically assessing” the odds during aggressive encounters and only engage in aggression when the odds are favorable or the resource value is high (McComb et al. 1994; Benson-Amram et al. 2011; see also Borrego 2017). Finally, elephants have extremely large-scale and long-lasting memories (Hart et al. 2008; Polansky et al. 2015), and elephants show olfactory discrimination at least equal to dogs (Arvidsson et al. 2012).

**Figure 1:**
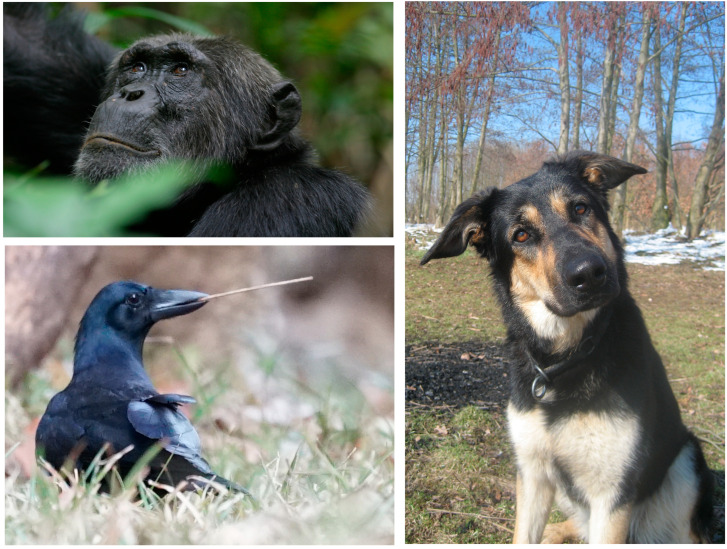
Clockwise from top left: chimpanzee, domestic dog, and New Caledonian crow participating in our research. Photographs by Simone Pika, Juliane Bräuer, and Natalie Uomini, respectively.

In addition to discoveries of surprising cognitive abilities in nonhumans, recent studies have also shown that animals that appear highly sophisticated in one cognitive domain often perform poorly in another. In the next paragraphs, we will illustrate this point by summarizing recent findings on three different, distantly related species on which we are experts (Figure 1): chimpanzees (*Pan troglodytes*), domestic dogs (*Canis familiaris*), and New Caledonian crows (*Corvus moneduloides*). Chimpanzees are one of two closest living relatives to humans, and due to this shared phylogenetic trajectory, they are expected to share many cognitive skills with humans. Dogs have a long domestication history with humans, in which they have evolved some special skills. New Caledonian crows show sophisticated tool manufacturing skills and provide an example of convergent evolution of cognitive skills (Hunt and Uomini 2016; see also below). These species are fairly well-studied, providing us with enough data to illustrate the main point of the present paper—that there is not always “one cognition”.

### 2.1. Chimpanzee Cognition

Chimpanzees have a very rich set of cognitive skills, and concerning the physical domain, often perform similarly to human children in captive settings (see, for example, Herrmann et al. 2007; Hanus et al. 2011). These findings renewed debates and theories about how human and chimpanzee cognitions differ (i.e., Tennie et al. 2009; Herrmann et al. 2007; Tomasello et al. 2005; Premack and Woodruff 1978; Kellogg and Kellogg 1933). Recent studies have shown that chimpanzees can also solve social problems using skills, such as mind-reading, that were previously thought to be uniquely human (i.e., Krachun et al. 2009; Call and Tomasello 2008). Chimpanzees seem to operate—at least on an implicit level—with an understanding of false beliefs, as they reliably look in anticipation of an agent acting on a location where an object is falsely believed to be hidden, even though the chimpanzees know that the object is no longer there (Krupenye et al. 2017, 2016; Kaminski et al. 2008). Moreover, chimpanzees are aware of others’ visual perspectives (Figure 1) to target information toward ignorant group members (Crockford et al. 2017; Crockford et al. 2012) and seem to plan for the future by building their night nests in the direction of their anticipated feeding tree the next morning (Janmaat et al. 2014). They communicate in referential ways and show similarities to human conversational turn-taking (Pika and Mitani 2006; Pika et al. 2018). In other social domains, chimpanzees also show remarkable behaviors comparable to humans; for example, they incur costs to watch the punishment of antisocial others (Mendes et al. 2018).

Thus, the findings on chimpanzee cognition strongly support the Social Intelligence Hypothesis as this species lives in fission–fusion societies and faces selection pressure for general cognitive flexibility to deal with frequent unpredictable situations. However, although they show great flexibility in many distinct tasks, chimpanzees do not outperform other species from less complex societies in *all* cognitive domains. For instance, chimpanzees are only able to use pointing cues to locate hidden food in competitive, rather than cooperative, experimental tasks (Bräuer et al. 2006; Herrmann and Tomasello 2006; Hare and Tomasello 2004). This limitation seems to be related to the fact that cooperative pointing does not play a dominant role in their social environment (Bräuer et al. 2006; Hare and Tomasello 2004; see above) and is instead restricted to specific contexts and social settings (e.g., Pika and Mitani 2006; Pika 2014). Another study showed that captive chimpanzees are outperformed in inhibition tasks by orangutans (*Pongo abelii)* (Vlamings et al. 2010) that live semi-solitary. This difference may be due to chimpanzees being strongly attracted by the food, as they face much stronger food competition with group mates in their natural environments than do orangutans (Vlamings et al. 2010; Pusey and Schroepfer-Walker 2013; Goodall 1986). Thus, although living in fission–fusion societies might have created selection pressures for the development of a number of cognitive skills in chimpanzees, other skills such as inhibition may have been less adaptive. Indeed, it is conceivable that the pressure to react quickly in social situations with high competition was more adaptive for chimpanzees than the development of inhibition skills. Future studies should focus more on socially and ecologically relevant needs for chimpanzees. Studies could, for example, address how the short reaction times of chimpanzees influence their behavior in rapidly-changing social interactions (Inoue and Matsuzawa 2007).

### 2.2. Dog Cognition

The domestic dog was domesticated about 30,000 years ago (Botigué et al. 2017; Thalmann et al. 2013) and shows outstanding skills in the social-cognitive domain (see Kaminski and Marshall-Pescini 2014; Miklosi 2007; Huber 2016 for reviews). These skills involve, in particular, the way dogs communicate with humans (Figure 1; Kaminski et al. 2004a; Kaminski et al. 2012; Kaminski et al. 2009a), their sensitivity to human attention and perspectives (Kaminski et al. 2017; Kaminski et al. 2009b; Call et al. 2003; Bräuer et al. 2013b), and their motivation to cooperate with humans (i.e., Bräuer et al. 2013a; Bräuer 2015; Piotti and Kaminski 2016; but see also Quervel-Chaumette et al. 2016; Marshall-Pescini et al. 2016). In contrast, dogs do not show exceptional physical cognitive skills but perform similarly to other nonprimate mammals and birds (Bräuer et al. 2006; Erdohegyi et al. 2007; Osthaus et al. 2005; Rooijakkers et al. 2009; Miletto Petrazzini and Wynne 2016). In some tasks, dogs are outperformed by wolves (*Canis lupus*), their closest living relatives, who are able to use causal cues to locate hidden food (Lampe et al. 2017). These findings have been explained as ancestral dogs experiencing selection pressures for cognitive flexibility only in the social domain, as they have adapted to function effectively in human society (Kaminski and Marshall-Pescini 2014). In comparison to wolves, dogs faced new challenges and thus may have acquired new social skills while losing those skills related to independent problem-solving and understanding their physical environment, skills that were critical for survival in the wild (Lampe et al. 2017). However, even within the social domain, there is evidence for wolves outperforming dogs. For instance, a number of studies showed that dogs cooperate poorly with each other (Bräuer et al. 2013c; Dale et al. 2016; Marshall-Pescini et al. 2017), suggesting that dogs were selected to cooperate *specifically with humans* (Bräuer et al. 2013a; Bräuer 2015; Range et al. 2019). In other words, the specific social environment of the domestic dog created a specific selection pressure for a specific cognitive skill such as the ability to cooperate *with humans*, but not for cooperative ability in general. This should be investigated further.

In sum, areas in which dogs show outstanding cognitive skills and outperform all other species—such as the ability to communicate with humans—are not necessarily linked together with other cognitive skills. Although dogs are more socially tolerant, more cooperative, and more sensitive to social cues than wolves, as predicted by the Domestication Hypothesis, the abovementioned findings go against predictions of the Social Intelligence Hypothesis or the Cooperative Breeding Hypothesis. Consequently, it is more likely that these abovementioned skills evolved *independently* from each other.

### 2.3. New Caledonian Crow Cognition

A disconnection between cognitive abilities has also been shown in a member of the corvid family. New Caledonian crows are renowned for their technological abilities (Weir et al. 2002; Taylor et al. 2007; Hunt and Gray 2004; Rutz et al. 2010; Hunt and Uomini 2016; Uomini and Hunt 2017). They not only use stick, stem, and grass tools in their natural environments (Figure 1) but also manufacture pandanus tools following templates to produce specific tool shapes that vary between populations and between individuals (Hunt and Gray 2004; Kenward et al. 2006; Taylor et al. 2012b). According to the Technical Intelligence Hypothesis (see above; Byrne 1997), one would predict that the New Caledonian crow, as a tool-making species, should possess enhanced physical cognition and should outperform closely related species in physical problem-solving tasks. However, evidence for enhanced abilities beyond their exceptional technological skills is still uncertain (see Taylor and Gray 2014 for a review). In the tool-using woodpecker finch (*Cactospiza pallida*), there was no difference in physical cognition between tool-using and non-tool-using individuals, when tested on non-tool physical tasks such as a movable perch that caused a food reward to be released or a puzzle box with a lid (Teschke et al. 2011). However, a follow-up study that tested woodpecker finches, New Caledonian crows, and related non-tool-using species from each clade found that non-tool-using small tree finches (*Camarhynchus parvulus*) performed equally to woodpecker finches. In contrast, New Caledonian crows outperformed carrion crows; the authors attribute these differing results to the more sophisticated tool skills of New Caledonian crows compared to woodpecker finches (Teschke et al. 2013). Therefore, it is possible that very elaborated tool skills and the related enhanced physical cognition can be found in New Caledonian crows. In particular, to resolve this question, observational data from wild crows are needed, because all of the data currently available on New Caledonian crows are from experiments in captive settings using artificial (human-created) tasks. In future studies, increased attention to observational data on wild crows can help to greatly improve ecological validity for this species, as called for by Kamil (1987), and as we discuss in Section 4.

Although New Caledonian crows may have a better understanding than related bird species of how to use metatools (i.e., the ability to use one tool on another tool; Taylor et al. 2007; Kenward et al. 2006; Gruber et al. 2019), they do not outperform them in other physical cognition tasks. So far, in captive setups, all corvid species tested have been found to show equal skills in the Aesop’s fable paradigm, a task designed to assess causal understanding of water displacement, in which subjects must discover how to drop stones into a water-filled container to raise the water level in order to obtain a floating reward (Logan et al. 2014), as well as in hook manufacture (Weir et al. 2002; Taylor et al. 2012b; Laumer et al. 2017), and trap-tube tasks (Taylor et al. 2009; Tebbich et al. 2007; Seed et al. 2006). In perceptual feedback studies, New Caledonian crows show the same understanding of the problem as keas (*Nestor notabilis*) and ravens, but they do not solve most string-pulling tasks as fully as keas and ravens, leading Taylor and colleagues (2010) to conclude that New Caledonian crows fail to perceive connectivity (Taylor et al. 2012a; Werdenich and Huber 2006; Taylor et al. 2010). The complex tool-using behaviors of New Caledonian crows also possibly do not enable them to make causal interventions, i.e., to learn a cause–effect relationship and then act to take advantage of that cause (Taylor et al. 2014; but see Jacobs et al. 2015 for an opposite viewpoint). Finally, these crows—although they can learn to produce collaborative behaviors to obtain food rewards in experimentally trained settings—do not understand the causality of cooperation, leading Jelbert et al. (2015) to conclude that the flexible use of physical tools does not necessarily enable animals to grasp that a conspecific can be used as a social tool (Jelbert et al. 2015). However, relatedness likely plays a role in the motivation to cooperate in this species, as New Caledonian crows are thought to spend most of their time in extended family groups (Holzhaider et al. 2011). If the crows understand that a conspecific can be used as a social tool only after direct experience with that individual, we would predict that the crows should be more likely to cooperate with kin than non-kin, particularly with kin who are already collaboration partners. To determine what factors underlie cooperative performance, future studies would need to test pairs of individuals of known relatedness, as well as to document the range of their cooperative behaviors in the wild.

Regarding other social cognitive skills, experimental results on social learning are consistent with the spontaneous behaviors documented in the wild. Logan and colleagues (2016) tested social transmission of various methods to open puzzle boxes and found that New Caledonian crows of all ages learned socially by stimulus enhancement (Logan et al. 2016). Similarly, long-term developmental observations in the wild showed that juvenile New Caledonian crows relied mostly on scaffolded individual learning—with templates as guidance to the final form of the tools—to develop their tool-making sequences; they produced the same tool types they saw being used and discarded by their parents, but they did not always produce the same variants as their parents (Holzhaider et al. 2010a; Holzhaider et al. 2010b). More developmental studies are needed to establish the variability and consistency in the social learning of New Caledonian crows (Uomini et al. 2020), but in comparison to other birds (i.e., Auersperg et al. 2014), so far they do not seem to perform especially well in social learning tasks.

In sum, the New Caledonian crow appears to be a species with excellent tool-related cognition but not outstanding social cognitive skills. However, it is not yet clear from the currently limited evidence if differences between New Caledonian crows and other species will become apparent when more nuanced tasks are used that more closely match the cognitive requirements of tool-use and tool-manufacture (Taylor and Gray 2014). At present, it appears that the selection pressures leading to the outstanding tool behaviors of New Caledonian crows did not foster the emergence of enhanced social skills (regarding cooperation and social learning), again suggesting that cognitive skills evolve in a domain-specific manner, often *independently* from each other (Macphail 1982).

## 3. When Animals Outperform Humans

As humans, our collective reluctance to acknowledge exceptional cognitive skills in nonhuman animals is at odds with the biological study of other species (i.e., Allen and Trestman 2017). It seems we are accustomed to accepting animal supremacy in anatomical features or physical performances: many mammalian species are bigger, stronger, or faster than humans, and we readily apply ecological explanations to determine such specific evolutionary adaptations. Interestingly, however, we do not appear willing to apply the same explanatory rigor in seeking specific selection pressures or species-typical ecological affordances when it comes to cognitive abilities. No physiologist would consider human respiration or digestion as a particularly useful reference point to describe or understand the variety and complexity of animal metabolism, but many psychologists still appear to mark human cognition as the pivotal point from which any comparison with nonhuman systems ought to start (Shettleworth 2010; Premack 2007; Vonk 2016). It is therefore not surprising that the term “cognition” (and “intelligence”, respectively), even in its broadest definition, is traditionally closely connected to “human cognition” (or “human intelligence”). As a result, we tend to overrate those cognitive skills that are human-like (see also Vonk and Povinelli 2012) and—what turns out to be scientifically more fatal—we run the risk of overlooking cognitive skills that play only a minor role or no role in human psychology. Furthermore, it is no surprise that we expect (and find) more similar forms of cognition in phylogenetically closely related species (i.e., Balda et al. 1996) and assume human-like clustering of cognitive abilities in other species. To be clear, we do not argue that cognitive abilities do not cluster at all in other taxa or species, but rather we doubt the presumption that they always cluster the way they do in humans. In this section, we review the outstanding skills of animals that sometimes outperform humans. These examples illustrate our point that humans cannot be considered “superior” to other animals, but rather that each species has its own cognitive specialisms, which may be unique or exceptionally elaborated within the animal kingdom.

In the following paragraphs, we describe just a few of countless examples of animal cognition that seem astonishing in comparison with human skills, but only when human cognitive performance is considered to be the highest possible level. However, in reality, these examples simply reflect species-typical behavioral repertoires.

Adult humans have been described as exceptionally patient, especially in contrast to other primate species, which are traditionally described as more impulsive and present-oriented (Tobin et al. 1996; Roberts 2002). However, recent work has shown that the assumed phylogenetic gap between human and nonhuman inhibitory skills is strongly influenced by specific parameters of the testing situation. Rosati and colleagues (2007), for instance, demonstrated that human patience drastically decreased when the relevant currency was food instead of money—as in the vast majority of previous human experiments—but also that chimpanzees and bonobos were much more patient than any other animal species tested so far. Chimpanzees also outperformed bonobos as well as human adults in a “food-waiting-paradigm” (Rosati et al. 2007). The intra-*Pan* difference is especially interesting as it directly relates to different ecological affordances of those two closely related species. Whereas wild chimpanzees have to deal with rather unpredictable and unstable fruit and meat resources (Wrangham et al. 1998), bonobos live in rather stable forest environments with comparatively stable and predictable food patches (Boesch et al. 2002; Furuichi et al. 1998). In particular, hunting and extractive technologies (e.g., nut-cracking)—two behavioral peculiarities of chimpanzees with major nutritional benefits—are rare or nonexistent in bonobos (Surbeck and Hohmann 2008; Boesch and Boesch-Achermann 2000; Mitani and Watts 2001). Habitat variation seems, therefore, a plausible explanation for the cognitive differences found between the two *Pan* species in the social and physical domains (Rosati et al. 2007; Rosati 2017; Rosati and Hare 2011). In sum, human patience is not as exceptional as previously thought.

Humans are also thought to be the most rational of all primates. Contrary to what is predicted by traditional economic models, however, humans are far from being rational maximizers when it comes to resource distribution in a social scenario. Instead of pure self-interest, their decision-making seems to be strongly affected by fairness concerns, which lead, in certain situations, to (seemingly) less rational decisions. Using the experimental paradigm of the Ultimatum Game, robust findings from several laboratories and multiple human cultures confirm the assumption that adult humans are willing to pay a cost by rejecting offers that they consider unfair (e.g., Fehr and Gächter 2002). In contrast, confronted with an adapted ape-version of the task, chimpanzees appear more rational than humans by accepting any non-zero offer and therefore maximizing their benefits more efficiently (Jensen et al. 2007). It is crucial to note that “rational” does not necessarily translate to “adaptive” here, even though it seems prima facie disadvantageous to prefer a zero outcome over a non-zero outcome just to punish a violation of a fairness principle. What appears rational and clever in the chimpanzee case is simply the most successful strategy in a social system that does not have strong other-regarding concerns of equality or fairness. In human societies, on the other hand, it might be highly adaptive to pay short-term costs to ensure fair future interactions. Such investment might pay off in the long run, given social systems with omnipresent implicit fairness expectations and explicit fairness norms.

Other than apes, several nonmammalian species show extraordinary cognitive performances that are comparable to those of human experts or even beyond. In order to navigate and communicate, birds have evolved considerable information-processing capabilities (McMillan et al. 2015). For example, many raptor bird species (e.g., eagles, hawks) are equipped with remarkable visual perception and classification abilities. Experiments with pigeons demonstrated that they can recognize different letters of the alphabet, can classify images based on animal taxa criteria (e.g., cats vs. dogs) or physical features of inanimate objects (chairs vs. tables), and are able to distinguish between Monet and Picasso paintings after some period of training (Watanabe 2001; Watanabe et al. 1995; Emery 2006). Results from a recent study even suggest that pigeons can be trained to “detect” cancer. The task consisted of classifying histopathological images as well as mammograms as either benign or malignant. Pigeons were able to generalize from training stimuli to new exemplars, and the performance level of the birds reached that of experienced human pathologists (Levenson et al. 2015). These skills seem impressive to a human observer, even though they demonstrate nothing but ordinary pattern recognition skills—a rather specific adaptation of pigeons. Similarly, it was shown that pigeons rely on a more efficient process than humans to visually identify objects presented in various spatial orientations. This difference is presumably rooted in the differing ecological demands placed on the visual systems of flying birds compared to earth-bound humans (Hollard and Delius 1982; McMillan et al. 2015).

Pigeons also outperform humans in other tasks. For instance, Herbranson (2012) compared pigeons and humans in a probability puzzle, i.e., the Monty Hall Dilemma. Subjects were given a choice from among three doors, one of which concealed a valuable prize. After an initial selection, one of the remaining, nonwinning doors was opened, and the participant was given a chance to switch to the other unopened door. The probability of winning is higher if the participant switches. Pigeons maximized their wins by switching on nearly all trials of a Monty Hall Dilemma analog, whereas humans utilized a suboptimal strategy involving probability matching (Herbranson 2012). One possible reason why humans used probability matching is that they were searching for a strategy that would be correct 100% of the time, whether or not that level of accuracy can actually be attained. This human use of probability matching might reflect an active search that progresses even when there are no consistent patterns to be found (Gaissmaier and Schooler 2008; Herbranson 2012). This idea is supported by another study in which rats and humans were trained in rule-based and information-integration category-learning tasks with visual stimuli. The generalization performance of rats and humans was equal in rule-based categorization, but rats outperformed humans on generalization in the information-integration task. While the performance of the rats was consistent with a nondimensional, similarity-based categorization strategy, humans again showed a bias toward rule-based strategies, which in that case impeded their performance on generalization tasks (Vermaercke et al. 2014).

Humans are also outperformed in spatial memory tasks, in particular by specific bird species. The most impressive spatial memory has been demonstrated by members of food-storing bird families like parrots and corvids (see Shettleworth 1983; for overviews, see Clayton et al. 2006; Clayton and Emery 2002). Some of these bird species (e.g., Clark’s nutcracker *Nucifraga columbiana* and marsh tit *Poecile palustris*) are able to remember over 100 cache sites after time delays of several months, by far exceeding the average human memory. Remarkably, not only can bird species that cache food for themselves (e.g., marsh tits) correctly remember the hiding locations they visited before, but so can species that are specialized in cache pilfering. Great tits, for example, are capable of memorizing caching locations just by “secretly” observing marsh tits caching (Urhan et al. 2017; Balda and Kamil 1992.)

What all these remarkable animal performances have in common is that they fascinate and puzzle human observers at the same time. The fascination comes from the fact that these demonstrated abilities are comparable or even superior to those of our own species within the same domains. They are puzzling because these are *cognitive* domains, and some aspects of cognition were traditionally thought to represent a unique characteristic of human minds that distinguishes us from other animals more than any other trait (Premack 2007; Uomini 2008; Shettleworth 2012; Uomini 2014; MacLean 2016; Uomini and Ruck 2018; Uomini et al. 2020). From an anthropocentric perspective, such skills would need an explanation because they challenge human superiority, but from a genuine biological perspective, such skills are simply examples of the unique traits that each species has evolved due to specific situations and needs. Just as physical traits (e.g., an elephant’s trunk, life history, or a digestive system that can process poisonous leaves) are considered in their evolutionary context, cognitive traits should similarly be considered according to their species-specific context.

## 4. Ecology, Perception, and Crucial Limitations

In addition to the fact that research approaches in animal cognition are often anthropocentric and driven by our own cognitive skills, we see another problem with the current comparative character of the field. The usual approach is to present the same task to different species. This strategy is problematic when the experimental task is not equally relevant to each of the compared species (Boesch 2007; Tomasello and Call 2008; MacLean et al. 2012; Roth et al. 2019). One classic example is the question of whether animals, chimpanzees in particular, can take the visual perspective of others. In a number of studies in which chimpanzees had to beg for food from humans, they did not show outstanding perspective-taking skills (Povinelli and Eddy 1996; Kaminski et al. 2004b). However, begging from humans is far from being a natural situation for a chimpanzee, which led Hare and colleagues to create a competitive situation in which two chimpanzees compete over a food resource. Given such a new and ecologically relevant situation, chimpanzees suddenly knew what others can see (Hare et al. 2000; Bräuer et al. 2007) and even understood what others have seen (i.e., that seeing leads to knowing; Hare et al. 2001; Hare 2001). Kaminski and colleagues (2008) followed up the idea of a competitive and, therefore, relevant situation and tested pairs of chimpanzees in a setup in which they sat opposite to each other and competed over two pieces of food on a sliding table that was pushed back and forth. They found that chimpanzees understood what their rival knows, but not what the rival believes (Kaminski et al. 2008). Thus, experiments do not have to be “natural”, but species-relevant, and subjects have to understand the test situation. Although subjects in these studies were not tested in a “natural” competitive situation in which chimpanzees physically compete over food, they showed very flexible perspective-taking skills. This finding suggests that it was crucial that chimpanzees were able *to perceive* the situation as competitive.

Certainly, one aspect of experiments that is often overlooked is how animals are able to perceive a situation. Shettleworth’s definition of cognition includes perception as it refers to adaptive information processing in the broadest sense. The modality in which a task is presented is absolutely crucial for detecting the cognitive potentials of a given species. For example, until now, nearly all studies about dog cognition have taken an anthropocentric view, mainly searching for skills and modalities that are important for humans, such as perspective-taking, cooperation, social learning, and visual or auditory communication. Most data on dogs’ understanding of their social and physical environment is based on performance in the visual or auditory modality (Bräuer et al. 2013b; Kaminski et al. 2004a; Kundey et al. 2010), even though olfaction is the most relevant sense that dogs use to explore their environment (Gazit and Terkel 2003; Horowitz et al. 2013). Therefore, dog cognition studies should, in fact, design tests based on olfaction, not on vision or hearing. Indeed, a recent study by Bräuer and Belger (2018) suggests that dogs have a flexible representation of what they smell. The fact that olfaction was neglected in dog cognition research until recently, when dogs were shown to excel at a task when it was reframed in the olfactory modality, illustrates how an anthropocentric approach can create the appearance of limitations in cognitive performance. On the contrary, apparent limitations can be due simply to the use of a perceptual modality that is disadvantageous to the animal being tested.

The modality also seems to be crucial in biological market tasks. In a study by Salwiczek and colleagues (2012), individuals of several fish and primate species had to make a choice between two actions in a foraging task. They could choose between two plates (differing in color and patterns to allow discrimination) with exactly the same food. However, one plate was ephemeral and the other one permanent. The food maximizing solution involved eating from the ephemeral food source first and only then from the permanent one that yielded an additional delayed reward. This task was ecologically relevant for the tested cleaner fish as it mimicked the simultaneous visit of a resident and a visitor to the cleaning station. Indeed, the cleaner fishes outperformed the tested chimpanzees, orangutans, and capuchin monkeys in that task (Salwiczek et al. 2012). However, when the task was made more perceivable for primates, primates improved their performance: when the food was colored instead of the plates, and when the food reward was hidden, capuchin monkeys readily learned to solve the task (Prétôt et al. 2016). Similarly, rats and pigeons could solve the task when there was a 20-second delay between the choice and its outcome (Zentall and Case 2018). These examples reflect the prime importance of perceptual modality on reaching conclusions about a species’ cognitive abilities.

In addition to the importance of modality on how species perceive tasks, there are ecological restrictions to what a member of a certain species can learn (Campbell et al. 2008). For example, cleaner fishes can only use generalized rule learning when the rule has ecological relevance. They can learn that predators are safe havens when chased by a punishing client while nonpredatory clients are not. However, they cannot learn to approach a nonpredatory client as a safe haven (Wismer et al. 2016). Similarly, bees can associate color with food but apparently not with danger (de Ibarra et al. 2014; Craig 1994). This difference between learnability in modalities might be comparable to the way humans who get food poisoning in a restaurant will develop an aversion to the food rather than to the person they went out with.

In sum, we emphasize again the importance of ecologically relevant experiments to uncover cognitive processes in nonhuman animals. As we illustrated above, many experiments that were designed anthropocentrically found negative results, which were then prematurely generalized to the species. The solution to this problem is that the tested skills *and* the experiments themselves should be ecologically valid. Ecological validity can be achieved by taking into account the importance of perception due to the modality of task presentation and the limitations for learning. One of the hardest tasks for animal cognition researchers in the coming years will be to design experiments that can detect the upper limits of animals’ abilities—a challenge that is especially difficult for us, as humans, in the case of nonhuman cognitive abilities that exceed anything we can imagine with our limited perception and cognition.

## 5. New Challenges in Animal Cognition

We began with a reminder of the fundamental but sometimes neglected call (Kamil 1987) for comparative research to take a biocentric view of cognition and to avoid common anthropocentric viewpoints (Allen 2017; Shettleworth 2010; Shettleworth 2012; Vonk 2016). We see three concrete weaknesses in current animal cognition research. Firstly, there is a widely shared conception that certain cognitive clusters found in humans, such as technical intelligence, are similarly organized in other animals, although there is no clear evidence for such similarities (Ducatez et al. 2015; see Section 2). Secondly, skills that are on par with those of humans have sometimes been overrated in humans and underrated in other species (i.e., Boesch et al. 2017). Therefore, species-specific cognitive skills (i.e., Kaminski and Marshall-Pescini 2014; McMillan et al. 2015) and findings of species that outperform humans on distinct tasks were sometimes overlooked or not tested, as it is difficult to find the appropriate experimental design (see Section 3). Thirdly, another element that should be strengthened is the importance of ecologically relevant experimental designs that consider perception and the limitations for learning in the tested species (Bates and Byrne 2007; De Waal 2016; see Section 4).

In addition to the concerns we have raised here, other researchers have highlighted the need to consider within-species variability (Barrett 2016), phylogenetic factors (to control for effects of shared descent; Balda et al. 1996), and social characteristics (e.g., level of competition and tolerance; Hare 2001; Fröhlich et al. 2016), as well as the application of different methodologies across species (Leavens et al. 2017). To remedy these issues, researchers interested in animal cognition should collaborate to test a wider variety of animal taxa rather than only the most common model species with presumably human-like cognitive abilities (i.e., Rowell 1999; Stanton et al. 2017). As animal cognition research is a truly interdisciplinary subject that appeals to researchers from distinct disciplines such as psychology, biology, anthropology, and neuroscience (e.g., Osiurak et al. 2020), we need to acknowledge the fact that they can and should complement each other. For example, it would be helpful for behavioral ecologists to include more cognitive research in their studies as they are experts on the ecology of a given species. On the other hand, psychologists who usually concentrate on the mechanism of a behavior could consider the ecological relevance and the phylogenetic history of their behavior of interest. Hence, observational investigations of the natural behavior of species and experimental studies should go hand in hand to enable detailed insights into the cognitive potential of a given species (Janmaat 2019).

In summary, taking together all of the old and new criticisms that we have identified for the future of animal cognition, we advocate that

1. Studies should be clear about which cognitive skill(s) they are testing and should not interpret evidence for one skill as automatically proving another, untested skill. Research should not assume that cognitive skills cluster the way they do in humans, but rather should start from the expectation of multiple cognitions until proven otherwise.
2. Studies should be based on detailed knowledge of the natural behavior and the ecological environment of the test species, so that it is possible to generate precise hypotheses about the species’ performance on a specific cognitive task (Bates and Byrne 2007).
3. Experimental settings should take into account social structures, developmental constraints, and preferred modality of the species under study (Bates and Byrne 2007; Roth et al. 2019).
4. Studies with nonhuman animals should no longer target *only* typically human cognitive skills such as tool-use, self-control, or social cooperation, but should also test skills in which humans might be outperformed by other animals, such as visual and odor perception, working memory, and reaction time (i.e., De Waal 2016; Bräuer and Belger 2018; Inoue and Matsuzawa 2007).
5. A holistic approach should be implemented to better integrate laboratory and fieldwork of behavioral ecologists, including the conducting of more rigorous observations and field experiments (Janmaat 2019; Boesch 2010; Bueno-Guerra and Amici 2018).
6. An even wider variety of animal taxa should be tested—starting with species that are as yet untested and under-represented in experiments—to gain a whole picture of cognition in the Animal kingdom (Vonk 2016; Roth et al. 2019).

Once research turns to the study of each species’ cognitive skills for its own sake (Allen 1998; Holekamp 2007; Borrego 2017), we will gain a more relevant perspective on animals’ cognitive skills that incorporates factors such as ecology, social environment, behavior, and development (Uomini 2008; Sewall 2015; González-Forero and Gardner 2018; Uomini et al. 2020), overlain onto the recognition that unique single cognitive capacities in some species coexist with full-blown cognitive arrays in others. Hence, there is not “one cognition”.

## Figures and Tables

**Table 1:** Citations of animal cognition papers from Web of Science, all databases 1894–2020 (accessed 25 April 2020), for the following search terms in the “topics” field: “animal cognition”, “animal psychology”, “cognitive ethology”, “comparative cognition”, “comparative psychology”.

Topic Search Term	Number of Publications	Total Citations	Total Excluding Self-Citations	Citations Per Year in 2000	Citations Per Year in 2019
animal cognition	1202	18,550	17,760		
animal psychology	435	3329	3295		
cognitive ethology	216	3242	3051		
comparative cognition	642	8379	7822		
comparative psychology	1376	13,496	12,587		
**Any of the above**	**3657**	**43,590**	**39,619**	**396**	**4394**
